# Plasmon-induced dual-wavelength operation in a Yb^3+^ laser

**DOI:** 10.1038/s41377-019-0125-2

**Published:** 2019-01-30

**Authors:** Laura Sánchez-García, Mariola O. Ramírez, Rosa Maria Solé, Joan J. Carvajal, Francesc Díaz, Luisa E. Bausá

**Affiliations:** 1 Deparment Física de Materiales, Instituto Nicolás Cabrera and Condensed Matter Physics Center (IFIMAC), Universidad Autónoma de Madrid, 28049 Madrid, Spain; 2 Universitat Rovira i Virgili, Departament Química Física i Inorgànica, Fisica i Cristal·lografia de Materials i Nanomaterials (FiCMA-FiCNA) - EMaS, E-43007 Tarragona, Spain

**Keywords:** Solid-state lasers, Nanophotonics and plasmonics

## Abstract

Expanding the functionalities of plasmon-assisted lasers is essential for emergent applications in nanoscience and nanotechnology. Here, we report on a novel ability of plasmonic structures to induce dual-wavelength lasing in the near-infrared region in a Yb^3+^ solid-state laser. By means of the effects of disordered plasmonic networks deposited on the surface of a Yb^3+^-doped nonlinear RTP crystal, room-temperature dual-wavelength lasing, with a frequency difference between the lines in the THz range, is realized. The dual-wavelength laser is produced by the simultaneous activation of two lasing channels, namely, an electronic- and a phonon-terminated laser transition. The latter is enabled by the out-of-plane field components that are generated by the plasmonic structures, which excite specific Raman modes. Additionally, multiline radiation at three different wavelengths is demonstrated in the visible spectral region via two self-frequency conversion processes, which occur in the vicinities of the plasmonic structures. The results demonstrate the potential of plasmonic nanostructures for inducing drastic modifications in the operational mode of a solid-state laser and hold promise for applications in a variety of fields, including multiplexing, precise spectroscopies, and THz radiation generation via a simple and cost-effective procedure.

## Nanoscale lasers with dual wavelengths

A dual-wavelength nano-laser can be achieved simply and cost-effectively by adding a disordered network of silver nanoparticles to an ytterbium-doped rubidium titanyle phosphate (RTP) crystal. Luisa Bausa of Universidad Autónoma de Madrid and colleagues in Spain found that this system led to the simultaneous generation of laser action at 1075 and 1052-nm wavelengths in the near-infrared range. This happens because the disordered networks of silver nanoparticles both enhance the excitation rate of the ytterbium ions and the vibration modes of the RTP crystal. The team also found that the emitted wavelengths can self-mix to produce multi-wavelength radiation in the visible green spectral region. The results demonstrate the potential of this sort of system for applications in multiplexing, precise spectroscopies, and the generation of terahertz radiation for medical imaging.

## Introduction

During the last decade, the unique properties of surface plasmon resonances for manipulating light at subwavelength volumes have been exploited to produce various types of plasmon-based lasers^1–3^. These systems provide subwavelength confined modes, which, together with the reduced physical device dimensions, hold promise for potential applications in ultradense data storage, ultracompact photonic circuits or extremely sensitive biodetection, among others^4–7^. The plasmon lasers that have been reported so far are based on a variety of configurations that combine gain media (mainly, dyes and semiconductors) with metallic nanostructures. Depending on the architecture, novel functionalities (such as an extreme field compression or highly directional laser action) can be realized^8–13^.

Recently, plasmon-assisted lasing at the nanoscale was reported in a Nd^3+^-doped solid-state laser (SSL) in association with linear chains of silver nanoparticles (NPs)^14^. In that system, the possibility of multiline operation has also been demonstrated. Namely, room-temperature near-infrared (NIR) lasing and, simultaneously, green and tunable blue radiation with subwavelength confinement, have been demonstrated in Nd^3+^:LiNbO_3_ crystal via various self-frequency-mixing processes at the metal-dielectric interfaces^15^. These results extend the inherent advantages of SSLs, such as their frequency stability, to subwavelength scales and pave the way for potential multifunctional operation of plasmon-assisted SSLs in applications that require multiplexing, which have yet to be explored at the nanoscale. In this context, the search for new designs that involve both rare-earth-based systems and plasmonic arrangements can lead to novel performances and functionalities, thereby further expanding the capabilities of these systems. In particular, the incorporation of Yb^3+^-based SSLs into the new class of plasmon lasers is of substantial interest, since it enables the exploitation of the interesting properties of Yb^3+^ ions at the nanoscale. Yb^3+^ is an attractive optically active ion that is relevant to a variety of fields^16^. Indeed, it can act as a NIR emitter for applications in bioimaging (its emission appears in the second biological window)^17^, as a sensitizer in energy transfer processes for displays^18^, in telecom devices^19^, in NIR-photocatalysis^20^, and in photovoltaics^21^.

As a laser ion, Yb^3+^ has several advantages over other rare-earth ions: It has a simple energy-level scheme that consists of two manifolds (^2^F_7*/*2_ ground state and ^2^F_5*/*2_ excited state) separated by an energy of ~10,000 cm^−1^; hence, Yb^3+^-doped systems are suitable for laser action in the 1.05–1.07 µm range. This ion also exhibits a low quantum defect and a high quantum efficiency; for the case of Yb^3+^-doped RbTiOPO_4_ (Yb:RTP), the quantum efficiency has been determined to be 81%^22^. Additionally, due to its 4*f*^13^ electronic configuration, its 4*f* electrons are less shielded than those of other Lanthanide ions. This leads to strong electron–phonon coupling and, hence, to the presence of vibronic or phonon-terminated transitions, which enable the realization of tunability and the generation of ultrashort pulses in the NIR region^23–25^.

In a previous work, the authors introduced a novel approach for enhancing the photoluminescence of Yb^3+^ ions in RTP crystal. As reported, silver nanoparticles connected in disordered plasmonic networks (DPNs) onto the surface of Yb^3+^-doped RTP produced a remarkable enhancement of the overall Yb^3+^ photoluminescence. This enhancement was mainly due to the increase of the Yb^3+^ excitation rate, which was attributed to the near-field distribution produced by the DPNs^26^.

Here, we demonstrate the possibility of a plasmon-induced dual-wavelength laser in the hybrid DPN-Yb^3+^:RTP system. The simultaneous oscillation of two laser lines appears as a consequence of the effect of the DPNs deposited on the surface of the Yb^3+^-doped RTP laser crystal. Under the conditions imposed in our experiment, in the absence of plasmonic nanostructures, lasing occurs exclusively at the ^2^F_5/2_(0’) → ^2^F_7/2_ (3) transition at 1075 nm. However, in the presence of DPNs, dual-wavelength lasing is realized, thereby allowing simultaneous laser action at 1075 and 1052 nm. The emission at 1052 nm is associated with a phonon-terminated transition that is preferentially enhanced by the DPNs, since these plasmonic structures produce not only the excitation rate enhancement of Yb^3+^ but also the selective enhancement of the Raman modes of the RTP crystal. Thus, by exploiting the near-field properties of the DPNs and the electron–phonon coupling of Yb^3+^ transitions, lasing of an additional line is induced by the effect of the plasmonic nanostructures in our SSL system, thereby yielding the dual-wavelength behavior.

Additionally, due to the quadratic nonlinear character of the RTP crystal, the obtained laser emissions at 1052 nm and 1075 nm are self-frequency mixed in the vicinities of the DPNs. As a result, multi-wavelength radiation is obtained not only in the NIR but also in the green spectral region at 526 nm, 537.5 nm, and 531.7 nm, due to various frequency mixing processes of the laser lines occurring in the vicinities of the DPNs.

The results demonstrate the high potential of plasmonic nanostructures for inducing drastic modifications and novel functionalities into the operational mode of SSLs, and hold promise for extending the relevant features of Yb^3+^ lasers to the nanoscale. Moreover, the demonstration of self-frequency mixing of the NIR-emitted lasing lines is of interest for potential applications in THz radiation generation, which could be realized via nonlinear self-frequency difference due to the high nonlinear coefficients of the RTP crystal and the field enhancement provided by the DPNs^27,28^. Wavelength division multiplexing, sensing, and precise spectroscopy are among the applications that could benefit from the effects of plasmonic nanostructures integrated into platforms based on SSLs^29–31^.

## Results

**Fig. 1:**
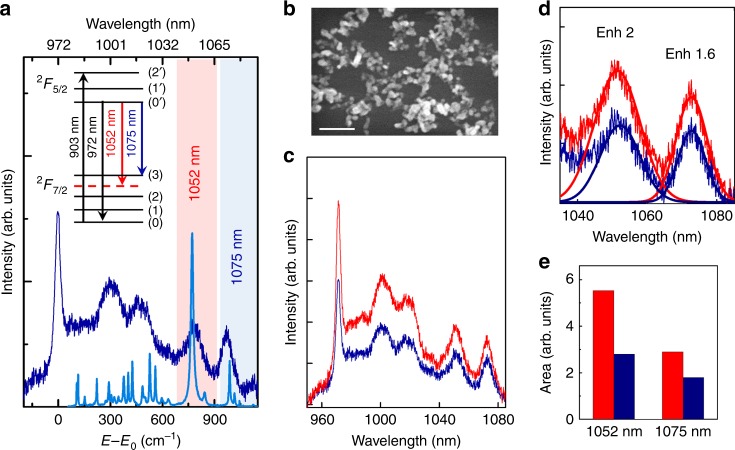
Plasmonic enhancement of Yb^3+^ luminescence. **a** Polarized room-temperature emission spectrum of Yb^3+^ in RTP (dark-blue spectrum) obtained for the geometrical configuration that is relevant for lasing experiments (the incident beam propagates along the *x* optical axis of RTP and the emitted light is polarized parallel to the *z*-axis). The emission spectrum is compared with the Raman spectrum of Yb^3+^:RTP obtained in the *z(xx)*$\bar z$ configuration (light-blue spectrum). The zero energy of the Yb^3+^ emission spectrum is located at the energy position of the ^2^F_5/2_(0’) → ^2^F_7/2_(0) electronic transition, which takes place at 972 nm (E_0_ = 10289 cm^−1^). The inset shows the energy-level scheme of Yb^3+^ ion in RTP. The black, blue, and red arrows correspond to the excitation transition at 903 nm, the ^2^F_5/2_(0’) → ^2^F_7/2_(3) emission at 1075 nm and the phonon-terminated transition at 1052 nm, respectively. **b** A representative SEM image of a DPN formed by the aggregation of Ag NPs. The scale bar corresponds to 500 nm. **c** Emission spectra of Yb^3+^ in RTP in the vicinities of an Ag DPN (red) and for bare Yb^3+^-doped RTP (blue). **d** Detail image of the photoluminescence spectra of Yb^3+^ in the presence (red) and absence (blue) of the Ag DPNs that shows the 1040–1080 nm region. The spectra include the Gaussian fits that were used to determine the enhancement factor of each emission band. **e** A bar diagram that represents the areas of the emission bands of Yb^3+^-doped RTP centered at 1052 nm and 1075 nm in the presence (red) and absence (blue) of the Ag DPNs

RTP is an orthorhombic biaxial crystal that belongs to the space group *Pna*2_1_, with point symmetry group *mm2* and crystallographic axes *a, b*, and *c*, that are parallel to the principal optical axes *x*, *y*, and *z*^32^. Figure 1a shows the room-temperature emission spectrum of Yb^3+^ in RTP (dark-blue line) obtained under excitation at the ^2^F_7/2_(0) → ^2^F_5/2_(2’) transition at 903 nm. The spectrum is obtained for the geometrical configuration that is relevant for lasing, namely, the incident beam propagates along the *x* optical axis of RTP and the emitted light is polarized parallel to the *z*-axis^22^. The spectrum is consistent with the C_1_ crystal-field symmetry of Yb^3+^ in RTP crystal, which splits the ^2^F_7/2_ and ^2^F_5/2_ states into four and three Kramer’s doublets, respectively^33^. A strong and sharp peak that is associated with the ^2^F_5/2_(0’) → ^2^F_7/2_(0) transition of Yb^3+^ ions at 972 nm is observed, together with a broadband structure at lower energy that has maxima at 1002 nm, 1023 nm, and 1075 nm. These maxima are related to transitions that involve the upper components of the split ^2^F_7/2_ ground state, namely, ^2^F_5/2_(0’) → ^2^F_7/2_ (1,2,3), which are broadened due to the electron–phonon interaction as commonly observed in Yb^3+^-doped crystals^34^. Figure 1a shows a comparison between the emission spectrum of Yb^3+^ and the Raman spectrum of the Yb^3+^:RTP crystal, which have been adjusted to the same energy scale. The zero energy of the Yb^3+^ emission spectrum is located at the energy position of the ^2^F_5/2_(0’) → ^2^F_7/2_(0) electronic transition, which takes place at 972 nm (E_0_ = 10289 cm^−1^). The comparison of both spectra shows the energy coincidence of the most intense Raman line at ~780 cm^−1^ and the Yb^3+^ emission band at 1052 nm (E_0_–E = 782 cm^−1^ in Fig. 1a), thereby revealing the phonon-terminated character of this emission band. The emission at 1052 nm can be attributed to a phonon-terminated transition, in which a photon of 1052-nm wavelength is emitted from the ^2^F_5/2_(0’) level simultaneously with a phonon with energy of ~780 cm^−1^. The Stark energy-level scheme^32,26^ and the phonon-terminated transition (dashed line) are illustrated in the inset of Fig. 1a. Here, we note that the incorporation of Yb^3+^ as a dopant in the RTP host crystal at the concentrations that are used in our work has a negligible effect on the Raman spectrum of RTP, which is dominated by the collective vibrational modes of the RTP host crystal.

Figure 1b shows a representative SEM image of the DPNs that are formed on the Yb^3+^:RTP surface. These plasmonic structures are composed by Ag NPs, with sizes in the range of 60–90 nm, connected in a disordered network-like arrangement. As previously demonstrated, these structures are of special interest because they support both localized and delocalized plasmonic modes, thereby providing spatially and spectrally broad field-enhancement regions, which efficiently overlap with the absorption of Yb^3+^ ions^26,35^. By depositing the DPNs on the surface of the Yb^3+^:RTP crystal, enhancement of the Yb^3+^ luminescence is obtained in the vicinities of the metallic structures. Figure 1c compares the emission spectrum that was recorded in the vicinities of the DPNs (red line) and the spectrum that was obtained from bare Yb^3+^-doped RTP (blue line). The main effect of the plasmonic structures is the overall enhancement of the luminescence. The average intensity enhancement, which is determined by comparing the integrated spectra in the presence and absence of DPNs, was ~1.7. According to previous results, the dominant mechanism for the photoluminescence enhancement of Yb^3+^ in RTP is the increase in the Yb^3+^ excitation rate that is produced by the DPNs. The obtained average enhancement value accords with the expected excitation rate intensification for this crystal configuration^25^.

Although the gross spectral shape of the Yb^3+^ emission is essentially preserved in the vicinities of the DPNs, a detailed inspection of the spectra reveals differences, particularly in the region of interest for lasing (1040–1080 nm). Figure 1d shows a detailed illustration of the effect of DPNs in that spectral region, which includes the ^2^F_5/2_(0’) → ^2^F_7/2_(3) transition at 1075 nm and the phonon-terminated emission at 1052 nm (see the energy-level scheme in Fig. 1a). In addition to the overall intensification effect, a change in the relative intensities of these two bands occurs in the vicinities of the plasmonic structures. The intensity of the emission at 1052 nm is enhanced by a factor of 2, which is ~ 20% larger than that of the emission at 1075 nm (1.6). Figure 1e shows a bar chart that represents the integrated emissions of both bands recorded in bare Yb^3+^:RTP and in the vicinities of DPNs. The difference between the intensifications of those bands is related to the different nature of the transitions.

**Fig. 2:**
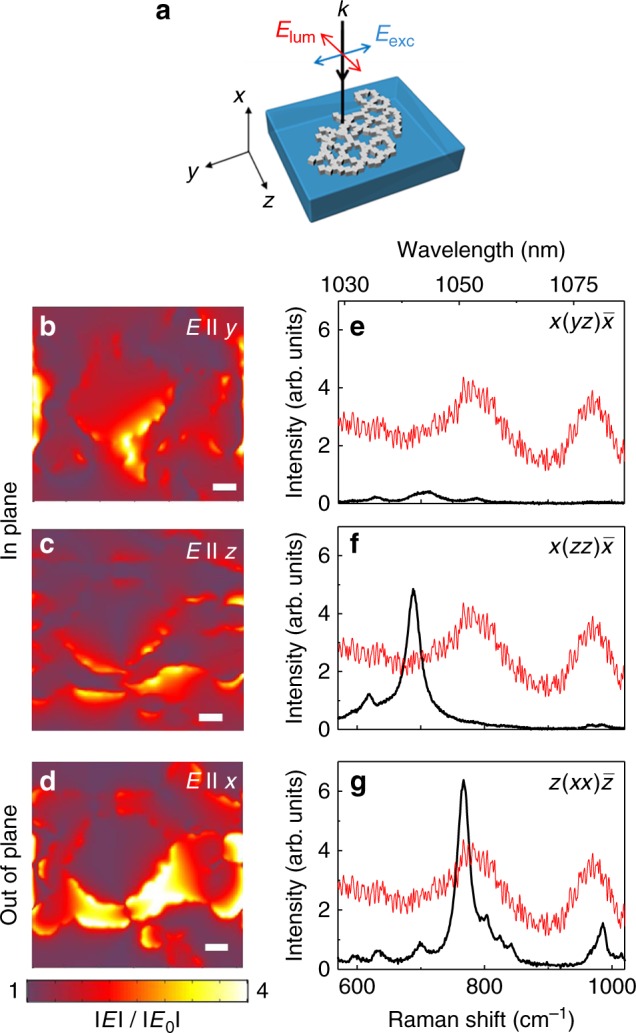
Near-field components generated by DPNs on Yb^3+^: RTP. **a** A sketch of the geometric configuration of the RTP crystal that was used in the PL and lasing experiments. **b**–**d** Near-field components of a DPN: (**b**) in plane component ***E*** II *y* (**c**) in plane component ***E*** II *z* and (**d**) out-of-plane component ***E*** II *x*. The white bar corresponds to 50 nm. **e**–**g** Raman spectra of Yb^3+^:RTP for various scattering configurations and orientations of the incident beam (black line). In panels **g**–**e**, the red line represents the emission spectrum of Yb^3+^:RTP in the lasing spectral region and for the configuration sketched in (**a**)

Accordingly, due to the phonon-terminated character of the 1052 nm emission, the effect of the near-field response of the DPN on the Raman spectra of Yb^3+^:RTP has been analyzed. Figure 2 shows the calculated near-field response of the Ag DPN at λ = 900 nm. The simulations have been performed by solving Maxwell’s equations in the time domain for an incident plane wave, namely, **E**_**0**,_ that is polarized along the *y*-axis and with its ***k*** vector directed along the *x*-axis (see Fig. 2a); the details are presented in Methods. Figure 2b, c, and d show the electric field components of the near-field response of the Ag DPN at λ = 900 nm, which are calculated in a plane located 5 nm below the RTP surface, where the DPN is deposited. As previously reported, the near-field shows broad spatial regions of field enhancement, together with hotspots, in accordance with the morphology and nature of the DPNs. Moreover, the DPNs generate additional polarization components to that of the incident field. This is observed in Fig. 2b, and c, which show the near-field that is associated with the in-plane field components, parallel (***E*** II *y*) and perpendicular (***E*** II *z*) to **E**_**0**_, respectively. They exhibit comparable near-field amplitudes. Stronger evidence of the generation of additional field components is provided by the appearance of an intense out-of-plane polarization component along the *x*-direction (***E*** II *x*), which corresponds to the largest values of local-field enhancement (Fig. 2d). The generation of the out-of-plane polarization component (along the *x***-**direction), which is not accessible in the far-field configuration, dominates the near-field amplitude. This is correlated with the preferential enhancement of the phonon-terminated transition at 1052 nm in the Yb^3+^ emission spectrum, which is due to changes in the excitation of the vibrational modes induced by the presence of additional field components generated by DPNs.

Figure 2e, f, and g show the high-frequency region of three representative Raman spectra of Yb^3+^:RTP crystal that were obtained in various scattering configurations. A portion of the spontaneous emission spectrum of Yb^3+^ in the lasing spectral region, which was obtained according to the configuration shown in Fig. 2a (the excitation polarization is parallel to the *y*-axis and the emission polarization parallel to the *z*-axis), has been superimposed with three Raman spectra by locating its zero energy at the ^2^F_5/2_(0’) → ^2^F_7/2_(0) electronic transition (see Fig. 1). Figure 2e, and f show the Raman spectra measured in the x-cut bare crystal for the *x*$(yz)\bar x$ and $x(zz)\bar x$ configurations defined according to Porto’s notation^36^ (see Methods). These configurations correspond to the B_2_ and A_1_ symmetry representations, respectively, and involve phonons that propagate along the [100]-direction. In particular, an intense peak at ~693 cm^−1^, which corresponds to a symmetric Ti–O stretching vibration, is observed in the $x(zz)\bar x$ configuration. However, the most intense peak of the high-frequency region appears at 780 cm^−1^ (Fig. 2g) in the polarized Raman spectrum belonging to the $z(xx)\bar z$ scattering configuration (A_1_ symmetry), which involves phonons that propagate along the [001]-direction^37^. This peak is related to the phonon-terminated Yb^3+^ transition observed at 1052 nm. In the crystal configuration of the lasing experiments, the DPNs deposited on the *y–z* surface generate the out-of-plane component, namely, ***E*** II *x*, which constitutes the major near-field contribution to the amplitude. This out-of-plane component enables the excitation of the vibrational modes of the $z(xx)\bar z$ configuration, not accessible without the presence of DPNs, with the subsequent enhancement of the phonon-terminated transition at 1052 nm.

**Fig. 3:**
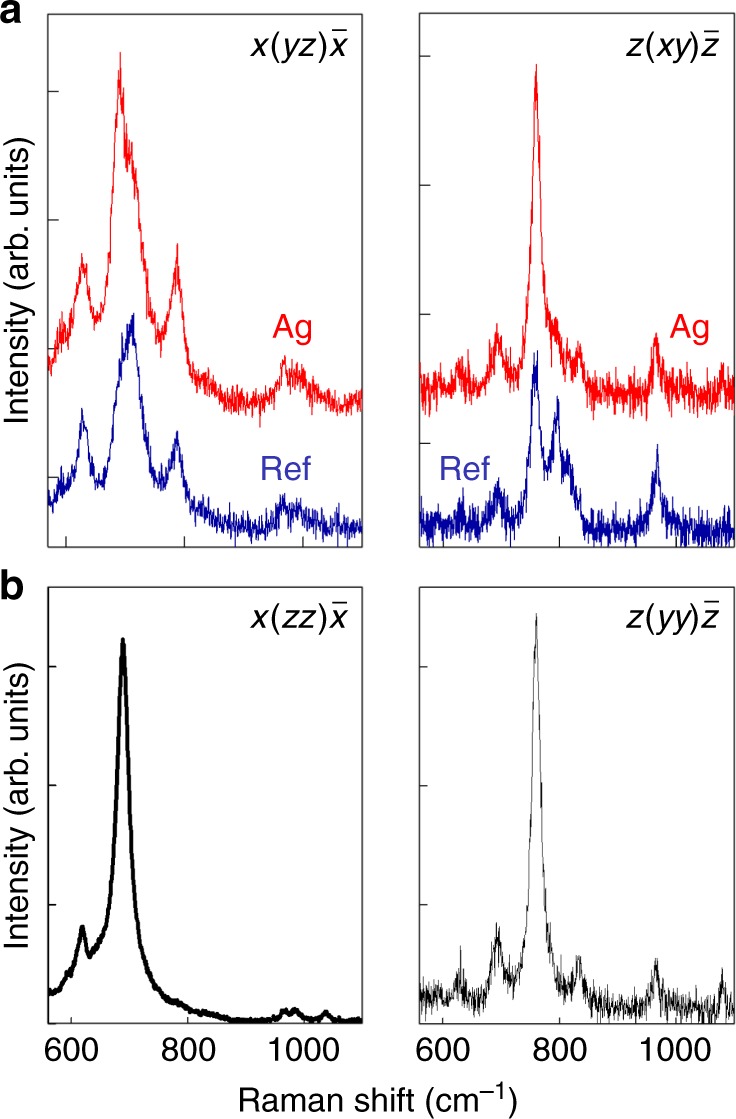
Effect of DPNs on the Raman spectra of RTP. **a** Raman spectra in the $x\left( {yz} \right)\bar x$ and $z\left( {xy} \right)\bar z$ configurations in the presence of Ag DPNs (red spectrum) and for bare Yb^3+^:RTP (blue spectrum). **b** Raman spectra for orthogonal polarizations of the incident beam that were obtained in bare Yb^3+^:RTP

Experimental confirmation of the generation of orthogonal field components and, hence, of the excitation of additional vibrational modes, was obtained by analyzing the effect of DPNs on the Raman spectra. Figure 3 shows the spectra of Yb^3+^:RTP in the vicinities of the Ag DPNs (red lines in Fig. 3a) and compares them to those of bare Yb^3+^:RTP (blue lines in Fig. 3a) for two different experimental configurations: $x(yz)\bar x$ and $z(xy)\bar z$. The Ag DPNs enhance the Raman scattering of the Yb^3+^:RTP crystal; however, more interestingly, they dramatically modify the line shape of the Raman scattering spectrum via the selective enhancement of various lines. The selectively enhanced lines correspond to those that dominate the Raman spectrum in an orthogonal excitation configuration. For instance, in the case of the $x(yz)\bar x$ configuration, the line at 693 cm^−1^ is selectively intensified under the presence of DPNs. According to Fig. 3b, this line dominates the Raman spectrum of the $x(zz)\bar x$ configuration of bare Yb^3+^:RTP; however, it is clearly excited in the $x(yz)\bar x$ configuration due to the extra field *z* polarization component induced by the presence of the DPNs. Similarly, the strong intensification of the line at 780 cm^−1^ in the $z(xy)\bar z$ spectra is related to the excitation of this mode by the presence of the additional *y* field component that is generated by the plasmonic structures, as it dominates the orthogonal $z(yy)\bar z$ Raman spectrum in Fig. 3b (right panel). Therefore, the preferential intensification of the 1052 nm line in the vicinities of the DPNs shown in the PL spectrum (Fig. 1d) can be associated with the generation of an additional out-of-plane component generated by the DPNs, which enables the excitation of the vibrational mode at 780 cm^−1^, thereby enhancing the phonon-terminated emission line at 1052 nm.

Once the effects of the Ag DPNs on the emission properties of the *x*-cut Yb^3+^:RTP crystal had been characterized, we evaluated their influence on the laser properties by placing the Ag DPNs/Yb^3+^:RTP crystal into a Fabry–Pérot cavity (Figure S1). To demonstrate the potential of the Ag DPNs, the cavity was formed by mirrors that exhibited higher losses at 1052 nm than at 1075 nm. The reflectance spectra of the mirrors are shown in Figure S2 of the Supplementary section.

**Fig. 4:**
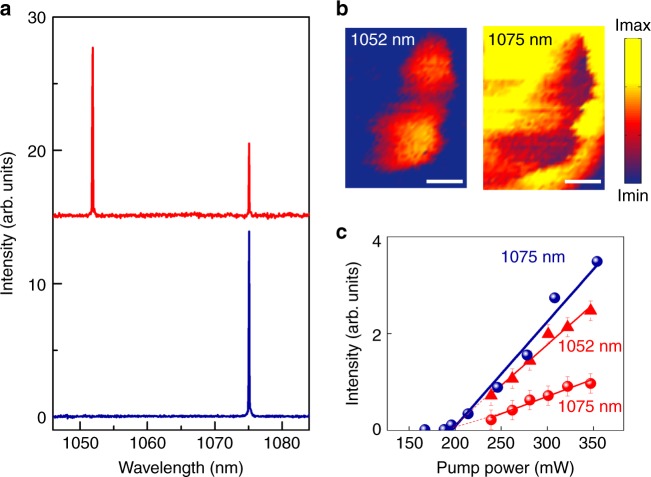
Plasmon-induced dual-wavelength lasing. **a** Room-temperature laser spectra collected in the vicinities of the Ag DPNs (red spectrum) and for bare Yb^3+^:RTP (blue spectrum). **b** Spatial distributions of the laser intensity for both laser wavelengths: 1052 nm (left) and 1075 nm (right). The dual-wavelength behavior is achieved at the central region, in the vicinity of a DPN. The scale bar corresponds to 5 microns. **c** Laser gain curves collected in the absence of DPNs (blue points) and in the presence of DPNs for the laser lines at 1052 nm (red triangles) and 1075 nm (red points)

Figure 4a shows the free-running laser spectra of Yb^3+^ in RTP recorded far from the metallic structures (blue line) and in the vicinities of the Ag DPNs (red line). In the absence of plasmonic structures, lasing is only obtained at 1075 nm and no laser action is observed at 1052 nm. However, in the vicinities of Ag DPNs, lasing is simultaneously obtained at both 1052 and 1075 nm (red lines in Fig. 4a). The plasmon-induced dual-wavelength lasing behavior can be related to the modification of the Yb^3+^ emission spectrum in the vicinities of the Ag DPNs. Recall that in the presence of the plasmonic structures, the vibronic line at 1052 nm is enhanced by a larger factor than that at 1075 nm, which allows overcoming the mirror losses at 1052 nm and, therefore, enables simultaneous lasing at both 1052 and 1075 nm. The spectral linewidth of the laser emission was measured to be ~0.7 cm^−1^ for both lines. According to the behavior of solid-state lasers, no change in the linewidths was observed above the threshold as the pump power was increased. To demonstrate the systematic behavior of the system, spatial maps of the lasing intensity as a function of the position on the surface for both 1052 nm and 1075 nm are shown in Fig. 4b. The dual-wavelength lasing occurs at the center of the image, which is correlated with the presence of a DPN. The dual-wavelength behavior is absent elsewhere in the crystal where there are no plasmonic structures, and only lasing at 1075 nm is achieved under our experimental conditions. On the other hand, the emergence of the 1052-nm line in the vicinity of a DPN is associated with the decrease of the intensity of the 1075 -nm line (Fig. 4b). Figure 4c shows the laser gain curve at 1075 nm in the absence of plasmonic structures (blue points) and the laser gain curves of the lines at 1052 nm and 1075 nm in the vicinities of the DPNs (red triangles and red points, respectively). A substantial reduction in the slope efficiency at 1075 nm is produced (by a factor of 2.3) to the benefit of the lasing gain at 1052 nm, when the system exhibits the dual-wavelength laser behavior. Since the initial level of laser action is the same for both lines, in the spatial region of overlap the gain should be redistributed between the two laser lines, which justifies the reduction in the lasing gain at 1075 nm to the benefit of the line at 1052 nm. In this sense, there is no competition between a near-field effect and a bulk effect, but rather different gain modes in the presence of DPNs, which confine the pump radiation at 903 nm to the nanoscale. In this case, the lines at both 1052 nm and 1075 nm originate from the same volume, as they are pumped by the same confined electric field at 903 nm.

**Fig. 5:**
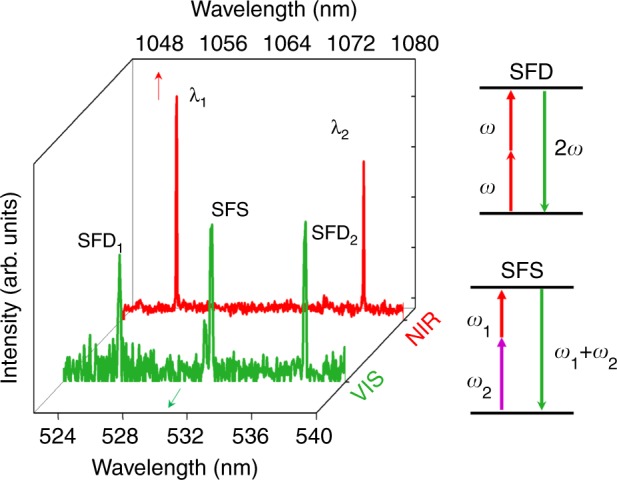
Multiline operation. NIR laser spectrum obtained in the vicinities of the Ag DPNs (red line) and green radiation collected in the same spatial region (green line) via the SFD and SFS processes. The arrow diagrams illustrate the frequency mixing processes via SFD at 526 nm and 537.5 nm, and via SFS of the NIR laser lines at 531.7 nm. The intensities of the frequency conversion (green) lines have been normalized to the intensity of the laser line at 1075 nm

Once dual-wavelength lasing had been demonstrated, multiline operation in the visible spectral region was achieved via two parametric frequency conversion processes in the vicinities of the DPNs: self-frequency doubling (SFD) and self-frequency summing (SFS) of the NIR laser lines. These parametric conversion processes occur due to the large second-order χ^(2)^ nonlinear response of the RTP crystal^27^. Figure 5 shows the spectra of the green radiation that were obtained in the vicinities of the Ag DPNs, along with schematic diagrams of the process. Three lines are generated at 526, 531.7, and 537.5 nm. The lines at 526 nm and 537.5 nm correspond to the SFD of the plasmon-assisted laser emission at 1052 nm and the SFD of the 1075 nm laser line, respectively. The line at 531.7 nm corresponds to the SFS of the 1052 nm and 1075 nm laser lines. The presence of the SFS line at 531.7 nm supports the plasmon-mediated dual lasing being spatially confined to the vicinities of the Ag DPNs, since for the SFS phenomenon to occur, both laser lines must occur at the same spatial location. For clarity, the intensities of the converted lines have been normalized to the intensity of the laser line at 1075 nm. Considering the wavelength dependence of the output coupler reflectivity and the experimental values, the frequency-converted green nonlinear lines were found to be four orders of magnitude less intense than the lasing radiation, which accords with the nonlinear self-frequency conversion efficiency of the intracavity lasing at NIR.

## Discussion

In this article, we have experimentally demonstrated plasmon-assisted dual-wavelength lasing operation from a Yb^3+^-based solid-state gain medium, on which Ag DPNs have been deposited. The dual-wavelength behavior is achieved in the vicinities of the DPNs, which induce lasing of the phonon-terminated Yb^3+^ line at 1052 nm, in addition to the 1075 nm line. The analysis of the photoluminescence and the Raman spectra for various configurations shows that the DPNs enable the excitation of the out-of-plane Raman mode that is involved in the phonon-terminated transition. As a result, selective enhancement of the 1052 nm laser transition is obtained at the vicinities of the metallic nanostructures, thereby leading to the simultaneous oscillation of two laser lines in the system. Compared with other sophisticated approaches that have been employed to realize dual-wavelength lasing from conventional rare-earth-based systems, the possibility of multi-wavelength lasing operation in the NIR range is demonstrated here via a simple and cost-effective procedure, thereby opening alternative routes for the development of plasmon-assisted multifunctional solid-state lasers. In addition, by taking advantage of the nonlinear character of the RTP ferroelectric host, we have demonstrated the possibility of extending the multi-wavelength operation from the NIR to the visible range. Multiline green radiation at 526, 531.7, and 537.5 nm is simultaneously achieved at the vicinities of the DPNs via two quadratic nonlinear frequency conversion processes (SFS and SFD) that involve the plasmon-induced laser line. The demonstration of self-frequency mixing of the NIR lasing lines further expands the potential of the system for multiplexing applications at various spectral ranges. Additionally, the suitability of the system for the self-frequency mixing process, along with the small frequency difference among the emitted wavelengths at the NIR, holds promise for the potential development of compact and low-cost plasmon-assisted coherent THz radiation sources, which have applications in many fields, including medical imaging, optical spectroscopies, communications, and security.

## Materials and methods

### Sample preparation

Yb^3+^-doped RTP single crystals were grown by the slow-cooling top-seeded solution growth (TSSG) technique, as previously reported^38^. During the crystal growth, Nb^5+^ was used as a codopant since it provides the charge compensation mechanism that is required when the trivalent Yb^3+^ ions substitute for the tetravalent Ti^4+^ ions in RTP, thereby enabling a higher Yb^3+^ concentration in the crystal^38^. The composition of the obtained crystals was RbTi_0.945_Nb_0.034_Yb_0.021_OPO_4_. For the lasing experiments, plates oriented with their main faces perpendicular to the *x* optical axis of RTP were cut. This crystal configuration allows access to the Yb^3+^ emission spectrum that is polarized parallel to the *z* optical axis, which exhibits the largest emission cross-section in the lasing spectral region^32^. The plate samples were of size 0.90 × 1.35 × 1.27 mm along the *x-*, *y-,* and *z*-axes.

Ag DPNs were directly formed on the surface of the Yb^3+^-doped RTP crystal via a photodeposition method. The samples were immersed in a 0.01 M AgNO_3_ solution at 50 °C and illuminated with above-bandgap UV light for 10 min. The UV illumination was carried out using a mercury lamp with its main line at 253.6 nm. Additional details on the DPN formation can be found elsewhere^28^. The SEM images of the Ag DPNs were obtained using a field emission gun electron microscope, namely, Philips XL30 Schottky.

### Spectroscopy experiments: Raman and photoluminescence

Photoluminescence (PL) and Raman spectroscopy experiments were conducted in a laser scanning confocal microscope. A 100x objective was used to focus the excitation on the Yb^3+^-doped RTP surface and to collect the backscattered emitted light. For the PL experiments, a Spectra Physics Ti:sapphire laser that was tuned at 903 nm was used as the excitation source. The excitation was polarized parallel to the *y* optical axis, and the emitted radiation was polarized parallel to the *z* optical axis. The emission was detected with a cooled InGaAs detector. The Raman experiments were conducted for various geometrical configurations by using the 488 nm line of an Ar^+^ laser. The Raman scattering was detected by a Peltier-cooled CCD detector. In both cases, spatially resolved spectra were obtained by scanning the surface of the crystal with an XY motorized stage with a spatial resolution of 0.3 µm. The results are reproducible, and no significant variations are observed when analyzing different regions of the crystal. Note that the DPNs are distributed onto the sample in areas of ~5 × 5 µm^2^ (Fig. 1c), and the diameter of the excitation spot is ~1 µm. Although the local-field components of the DPNs exhibit irregularities and hotspots on the nanoscale, the PL and Raman scattering spectra are collected in the far-field; hence, the results are averaged over a relatively large portion of a DPN structure.

The Raman scattering configurations were represented according to Porto’s notation *a(bc)d*, where *a* and *d* correspond to the propagation directions of the incident and scattered light, respectively, and *b* and *c* correspond to the polarization directions of the incident and scattered light, respectively.

### Laser experiments

A schematic diagram of the configuration of the lasing experiments is shown in Figure S1. Spatially resolved laser experiments were conducted by placing the Ag DPNs/Yb^3+^-doped RTP crystal into a Fabry–Pérot cavity. The cavity was positioned on a two-axis *xy* motorized platform with 0.3 µm spatial resolution. A CW Ti:sapphire laser (Spectra Physics) that was tuned at 903 nm, which corresponds to the ^2^F_7/2_(0) → ^2^F_5/2_(2’) transition of Yb^3+^, was used for optical pumping. A 10x microscope objective was used to focus the pump radiation and to collect the laser emission in backscattering geometry. The optical pumping was performed along the *x*-axis with the light beam polarized to the *y* optical axis. The laser emission was parallel to the *z* optical axis. The Fabry–Pérot resonator was composed of two plane-parallel mirrors that exhibited high reflection at the laser wavelengths (99.2% at 1052 nm and 99.7% at 1075 nm) and 92.3% transmittance at the pump wavelength; see Figure S2 in the Supplementary section. These values were carefully selected to prevent lasing at 1052 nm in the absence of DPNs, such that the effect of the plasmonic structures on the induced dual-wavelength lasing could be clearly evidenced. The laser radiation at 1052 nm and 1075 nm and the SFD and SFS radiation in the green spectral region were collected in the backscattering geometry and detected by a Horiba iHR 550 monochromator connected to a Horiba Synapse CCD. The lasing experiments were conducted at room temperature.

### Near-field simulations

The near-field distribution generated by the DPNs was obtained by solving Maxwell’s equations in the time domain. A commercial finite-difference time-domain (FDTD) software (Lumerical Solution) was used for the calculations. The DPNs were modeled by arranging Ag NPs with size ranging from 60–90 nm in a network-like arrangement that mimics the morphology of the DPNs that was observed in the SEM images. Those structures were simulated on top of an RTP substrate, which was modeled as a dielectric medium with a refractive index of 1.8.

The computational domain was discretized using a grid spacing of 0.8 nm. A plane wave that was directed perpendicular to the RTP/air interface was used for excitation. Perfectly matched layers were employed as boundary conditions in the planes that were perpendicular to the propagation of the plane wave and periodic boundary conditions for the remaining boundaries. The dielectric function of Ag was obtained by fitting the experimental data from Palik^39^. The refractive index of the RTP substrate was fixed at *n* = 1.8, according to the literature^40^.

## Supplementary information

Supplementary information
