# Supplementary material for: Plasmon-induced dual-wavelength operation in a Yb3+ laser
Source: Light Sci Appl. 2019 Jan 30;8:14. doi: 10.1038/s41377-019-0125-2 (PMC6351591; doi:10.1038/s41377-019-0125-2)
Supplement: Supplementary file 1 — Supplementary information [file 41377_2019_125_MOESM1_ESM.docx]

**Supplementary Information**

**Plasmon-induced dual wavelength operation in an Yb^3+^ laser**

Laura Sánchez-García^1^, Mariola O Ramírez^1^, Rosa Maria Sole^2^, Joan J. Carvajal^2^, Francesc Diaz^2^, Luisa E. Bausá^1*^

^1^ Dept. Física de Materiales, Instituto Nicolás Cabrera and Condensed Matter Physics Center (IFIMAC), Universidad Autónoma de Madrid, 28049 Madrid, Spain.

^2^ Universitat Rovira i Virgili, Departament Química Física i Inorgànica, Fisica i Cristal·lografia de Materials i Nanomaterials (FiCMA-FiCNA) - EMaS E-43007 Tarragona, Spain

**Experimental Set-up**

**Figure S1:** Schematic of the confocal microscope employed for the lasing experiments. The sample is placed inside a Fabry-Perot laser cavity mounted on a XY piezoelectric stage. As pump source, a CW Ti-sapphire laser tuned at 903 nm is directed to the sample using a ×10 magnification objective. The lasing signal is collected in reflection mode by the same objective and directed with a dichroic fielter (DF) to a monochromator equipped with a nitrogen cooled InGaAs detector.. The cavity length is equal to 1 mm and the Yb^3+^:RTP crystal thickness is 0.9 mm. The crystallographic axis are also shown in the figure. The optical pumping is performed along the *x* axis with the light beam polarized to the *y* optical axis. The dual wavelength lasing operation is polarized parallel to the *z* optical axis.

**Transmittance of the mirrors forming the Fabry-Pérot cavity**


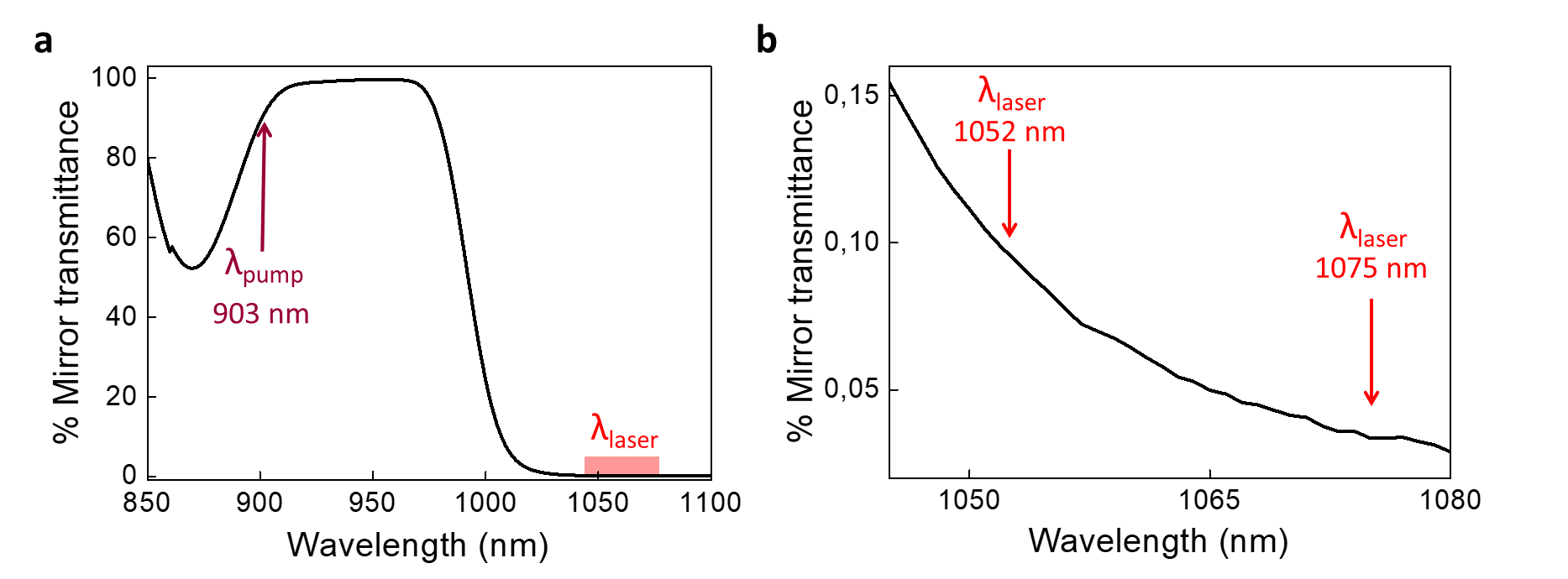


**Figure S2. (a)** Transmittance spectrum of the mirrors forming the laser cavity in the 850-1100 nm region. The arrow indicates the pump wavelength used in the experiments (903 nm). The shaded region marks the spectral region of interest for lasing. (**b)** Detail of the transmittance spectrum of the mirrors in the 1050 – 1080 nm region. The arrows mark the laser wavelengths at 1052 nm and 1075 nm.
